# Spatially Resolved Metabolomics and Network Pharmacology Reveal Extract D Nephrotoxicity Mechanisms in *Pleuropterus multiflorus* Thunb.

**DOI:** 10.3390/toxics13030182

**Published:** 2025-02-28

**Authors:** Haiyan Jiang, Ying Wang, Xiaoyan Duan, Shushu Guo, Xiaoyu Fan, Tianyu Zhou, Jie Li, Jiuming He, Jianbo Yang, Hongtao Jin

**Affiliations:** 1 New Drug Safety Evaluation Center, Institute of Materia Medica, Chinese Academy of Medical Sciences & Peking Union Medical College, Beijing 100050, China; jhystu@imm.ac.cn (H.J.); duanxiaoyan@imm.ac.cn (X.D.); fxykarma@imm.ac.cn (X.F.); lijie@imm.ac.cn (J.L.); 2 National Institutes for Food and Drug Control, Beijing 102629, China; wangying17@nifdc.org.cn; 3 Department of Life Sciences and Biopharmaceutics, Shenyang Pharmaceutical University, Shenyang 110016, China; ssguo918@163.com; 4 College of Pharmacy, Shaanxi University of Traditional Chinese Medicine, Xianyang 712046, China; zhoutianyu0620@163.com; 5 State Key Laboratory of Bioactive Substance and Function of Natural Medicines, Institute of Materia Medica, Chinese Academy of Medical Sciences & Peking Union Medical College, Beijing 100050, China; hejiuming@imm.ac.cn; 6 NMPA Key Laboratory for Safety Research and Evaluation of Innovative Drug, Beijing 102206, China; 7 Beijing Union-Genius Pharmaceutical Technology Development Co., Ltd., Beijing 100176, China

**Keywords:** *Pleuropterus multiflorus* (Thunb.) Nakai, spatially resolved metabolomics, renal toxicity, network pharmacology, mass spectrometry imaging

## Abstract

As a traditional Chinese medicine, the adverse hepatotoxicity effects of *Pleuropterus multiflorus* (Thunb.) Nakai (PM) have been documented. However, nephrotoxicity has been neglected as studies related to kidney toxicity mechanisms are limited. Our previous research reported that extract D [95% ethanol (EtOH) elution, PM-D] in a 70% EtOH PM extract showed more significant hepatotoxicity than other extracts. In the current study, PM-D was continuously administered to mice for 7 days at a dose of 2 g/kg (equivalent to a human dose of 219.8 mg/kg), which increased renal biochemical indexes and caused pathological kidney injury, suggesting renal toxicity. Therefore, network pharmacology and spatially resolved metabolomics were conducted to explore nephrotoxicity mechanisms underpinning PM-D. Network pharmacology indicated that *BCL2*, *HSP90*, *ESR1,* and *CTNNB1* genes were core targets, while the phosphoinositide 3-kinase (PI3K)/protein kinase B(AKT)/signaling pathway was significantly enriched. Spatially resolved metabolomics indicated heterogeneous metabolite distribution in the kidney, further indicating that PM-D nephrotoxic metabolic pathways were enriched for α-linolenic acid and linoleic acid metabolism, pyrimidine metabolism, carnitine synthesis, and branched-chain fatty acid oxidation. Our comprehensive analyses highlighted that nephrotoxicity mechanisms were related to oxidative stress and apoptosis induced by disordered energy metabolism, lipid metabolism issues, and imbalanced nucleotide metabolism, which provide a platform for further research into PM nephrotoxicity mechanisms.

## 1. Introduction

As a traditional Chinese medicine, *Pleuropterus multiflorus* (Thunb.) Nakai (Heshouwu, PM), a member of the Polygonaceae family, is divided into raw and processed products. Raw PM products expel toxins, eliminate carbuncles, eradicate malaria, lubricate the intestines, and assist with defecation. Processed PM products nourish the liver, kidney, and blood, strengthen muscles and bones, blacken hair, and reduce lipids [1]. PM has a wide range of medicinal properties, but reports of its adverse effects are increasing; PM hepatotoxicity is often reported in clinics, but the kidney burden caused by long-term usage cannot be ignored [2,3,4].

Relatively few studies have examined PM nephrotoxicity; therefore, nephrotoxicity mechanisms remain largely unclear. The long-term administration of PM at a dose of 0.6 g/kg in rats (equivalent to a dose of 0.1 g/kg in humans) caused kidney injury and induced renal cell apoptosis [5]. In in vivo studies, emodin-8-O-β-D-glucoside and emodin-type monoanthranone (PM components) appeared to cause histopathological kidney changes in Sprague Dawley rats; the former causing tubular hyaline droplet formation and the latter inducing tubular pigmentation and aggravating tubular basophilia [6]. Another study reported that three PM components (rhein, physcion, and emodin) exerted changes in blood urea nitrogen levels in mice after 14 days of oral administration [7]. In in vitro studies, emodin (PM monomer) exerted apoptotic/necrotic effects on human proximal renal tubular epithelial cells in a dose- and time-dependent manner [8]. A human renal cortical proximal tubule epithelial cell line (HK-2 cells) was used to screen for major anthraquinone in PM, after which, cells were significantly apoptotic upon emodin, aloe–emodin, and rhein administration [9]. In a metabolomics analysis of rat urine samples, liver and chronic kidney injury occurred due to PM, indicating that phenylalanine and tyrosine metabolic pathways were primarily disrupted [10]. Therefore, renal PM toxicity is a serious issue that requires more research.

In our previous work, the acute toxicity effects of different PM extracts and components were assessed in zebrafish embryos and showed that extract D toxicity [95% ethanol (EtOH) elution] in a 70% EtOH PM extract (PM-D) was considerably higher than other extracts. The 27 chemical components of PM-D were isolated and identified, including anthraquinones, polyphenols, anthraquinones, dianthrones, and seneciosides [11,12]. In acute toxicity tests, mice were orally administered a non-lethal PM-D dose (2 g/kg) for 7 consecutive days; PM-D not only increased aspartate aminotransferase (AST) and alanine transaminase (ALT) levels but also increased related liver pathology indicators, including liver cell necrosis and damage [13], and caused biochemical and pathological kidney damage. Thus, PM-D nephrotoxicity mechanisms in mice require further study.

Systematic research strategies are widely used to study multi-targeted toxicity mechanisms underpinning Chinese herbal extracts. Network pharmacology is based on a “disease–target–drug” interaction network which effectively predicts the multi-pathway regulation of different drugs in the body [14,15]. Spatially resolved metabolomics derived from mass spectrometry imaging provides in situ metabolite information, which reflects metabolic profile changes in different microregions of heterogeneous organs and uncovers metabolic regulatory network of drugs on the organism [8,9]. We previously integrated these approaches to investigate PM-D hepatotoxicity mechanisms and observed that PM-D-induced hepatotoxicity was closely related to cholestasis, mitochondrial damage, oxidative stress, and energy and lipid metabolism disorders [13]. Therefore, in this study, we integrated these techniques to examine PM-D effects on endogenous metabolites in mouse kidney microregions, identify PM-D nephrotoxicity-related biomarkers and potential nephrotoxicity mechanisms, explore correlations with hepatotoxicity mechanisms, and provide a reference for the safe clinical use of PM.

## 2. Materials and Methods

### 2.1. Plant Materials

PM was collected in Deqing, Guangdong province, China. It was preserved at the Research and Inspection Center of Traditional Chinese Medicine and Ethnomedicine, National Institutes for Food and Drug Control, State Food and Drug Administration, Beijing, China (No. 060104), after identification by associate Professor Ji Zhang (Research and Inspection Center of Traditional Chinese Medicine and Ethnomedicine, National Institutes for Food and Drug Control, State Food and Drug Administration) [11].

The 209.4 g PM powder was ground and subjected to extraction with ethanol. The extraction process was carried out three times in an ultrasonic water bath, with 200 mL, 100 mL, and 100 mL of 70% ethanol used, respectively, each time for 30 min. The combined extractions were condensed and lyophilized to obtain 40.0 g of freeze-dried powder. Then, freeze-dried powder was mixed with 100 mL water to form a suspension, which was filtered through a macroporous resin (DM-8, 1000 mL). After that, PM-D (1.9 g) was obtained by gradient elution in 95% ethanol and water [13].

### 2.2. Chemical Reagents

Na-carboxymethyl cellulose (CMC-Na) was obtained from Xilong Chemical (Shantou, China); high-performance liquid chromatography (HPLC)-grade acetonitrile and methanol were purchased from Merck (Darmstadt, Germany); ultrapure water was provided by Wahaha (Hangzhou, China); and the cryo-gel embedding medium was provided by Leica (Amsterdam, The Netherlands) [13].

### 2.3. Serum and Kidney Sample Collection

Male ICR mice (18–22 g) were acquired from Charles River Laboratory Animal Technology [License number: SCXK-(Jing) 2016-0006, Beijing, China]. Control mice were given 0.5% CMC-Na and the drug administration group received PM-D (2 g/kg) for 7 consecutive days, after which kidney tissues and serum samples were collected [13]. Kidneys were fixed in 4% paraformaldehyde, while kidney samples for mass spectrometry imaging were stored at −80 °C (*n* = 6). Animal studies were approved by the Animal Welfare Ethics Committee of the New Drug Safety Evaluation Center, Institute of Materia Medica, Chinese Academy of Medical Sciences, and Peking Union Medical College (No. 0000268).

### 2.4. Serum Biochemistry and Kidney Pathology Analyses

Serum creatinine (CREA) and urea levels were determined using a Beckman AU480 automatic biochemical analyzer (Brea, CA, USA). Kidneys fixed in 4% paraformaldehyde were embedded in paraffin wax and 4 μm thick sections prepared in a Leica RM2016 cryostat (Wetzlar, Germany). Sections were then stained with hematoxylin and eosin (H&E).

### 2.5. Network Pharmacological Analysis

Disease target data were collected from GeneCards (https://www.genecards.org/, accessed on 15 December 2023), Online Mendelian Inheritance in Man (https://omim.org/, accessed on 15 December 2023), and Comparative Toxicogenomics Databases (https://ctdbase.org/, accessed on 15 December 2023) using “renal toxicity” and “nephrotoxicity” as keywords. PM-D-related targets were obtained from the Swiss Target Prediction database (http://www.swisstargetprediction.ch/, accessed on 16 December 2023) based on the chemical component structures identified in PM-D. The overlapping targets of PM-D and nephrotoxic diseases were the targets of potential nephrotoxicity of PM-D. PM-D components and nephrotoxicity targets were imported into Cytoscape 3.8.2 to construct a “drug component–target–disease” network, where green nodes represent drug components, blue nodes represent targets, and orange nodes represent diseases, and edges represent the specific relationship between the two nodes. The degree value of a node represents the number of edges that a particular node has with other nodes. The protein–protein interaction (PPI) network was constructed based on the top 20 targets ranked by degree value in the “drug component–target–disease” network. The top 20 core targets were uploaded to STRING (https:/cn.string-db.org, accessed on 19 December 2023). The protein interaction strength was set to 0.4 to obtain information on interactions between the targets and further construct the PPI network. The PPI network was visualized by Cytoscape 3.8.2, where proteins were characterized by nodes and interactions between proteins were characterized by edges. The degree value of a node refers to the number of edges directly connected to that node. The nodes were colored with degree values; the darker the node color, the higher the degree value, indicating that the target protein interacts with more target proteins. Gene Ontology (GO) enrichment analysis and Kyoto Encyclopedia of Genes and Genomes (KEGG) pathway enrichment analyses were performed on all targets using DAVID (https://david.ncifcrf.gov/, accessed on 22 December 2023).

### 2.6. Molecular Docking

Molecular docking was used to verify key targets. Two core components (D11 and D12) were searched in PubChem. Three-dimensional structures of proteins were obtained from Protein Data Bank (https://www.rcsb.org/, accessed on 31 January 2025). Interactions between compounds and key proteins were simulated by Autodock 1.5.6. The results were imported into PyMol 2.5 for visualization.

### 2.7. AFADESI-MSI Data Acquisition and Analysis

#### 2.7.1. Sample Preparation

Frozen kidney tissue was fixed on a CM1860 cryostat (Leica Microsystems, Wetzlar, Germany) and thin 12 μm slices generated at −20 °C. Slices were then adhered to a positive charge desorption plate. After drying at −20 °C for 2 h and at room temperature for 1 h, sections were scanned to generate optical tissue images using a PrimeHisto XE scanner (MICROTEK, Shanghai, China).

#### 2.7.2. Collection Conditions

An air-flow-assisted desorption electrospray ionization (AFADESI)–mass spectrometry imaging (MSI) system consisted of an AFADESI ambient ion source and a Q-Orbitrap mass spectrometer (Q-Exactive, Thermo Fisher Scientific, Waltham, MA, USA). AFADESI was performed in positive and negative ion modes under the following operating parameters: spray voltage = 7000 V; transfer tube voltage = 3000 V; spray solvent = acetonitrile/water (8:2, *v*/*v*, 5 mL/min); spray gas pressure = 0.7 MPa; nitrogen pressure = 0.5 MPa; extracting gas flow = 45 L/min; *X*-axis scanning speed = 0.2 mm/s; *Y*-axis stepping distance = 0.2 mm; scan range = *m/z* 70~1000; capillary temperature = 350 °C; and resolution = 70,000.

#### 2.7.3. Data Processing

Mass spectrometry data were imported into MassImager 2.0 (Chemmind, Beijing, China) for image reconstruction, background deduction, and data extraction from tissue microregions. The sample dataset was further subjected to peak alignment, isotope removal, and normalization using Markerview™ software 1.2.1 (AB SCIEX, Milwaukee, WI, USA). Peaks with more than 50% of missing values in the sample were removed, and the unfiltered missing values were filled with the minimum value within the group. Subsequently, multivariate analysis was performed using SIMCA-P 15.0 (Umetrics, Umea, Sweden). Differential ions were screened using projected variable importance (VIP) >1, *p* < 0.05, and fold change in difference (FC) >2 or <0.5 as indicators. Based on precise relative molecular masses, differential ions were matched in the AFADESI-MSI metabolite database and identified in Human Metabolome Database (HMDB) (https://hmdb.ca/, accessed on 2 March 2024) and Lipid Maps (https://www.lipidmaps.org/, accessed on 2 March 2024) databases. Metabolic pathway enrichment analysis was performed in MetaboAnalyst 5.0 (https://www.metaboanalyst.ca/, accessed on 20 March 2024). Statistical processing was performed in GraphPad Prism 7.0 and Excel 2010 software. Differences between groups were tested using two-tailed unpaired Student’s *t*-tests. *p* < 0.05 was considered statistically significant.

## 3. Results

### 3.1. Effects of PM-D on Kidney in Mice

**Figure 1:**
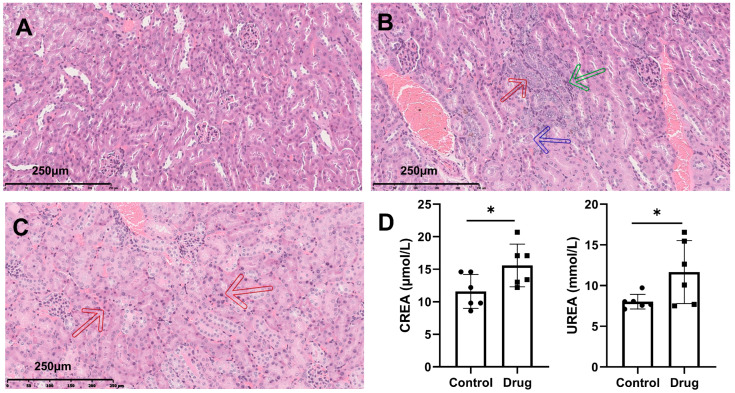
Effects of extract D from *Pleuropterus multiflorus* (PM-D) administration on renal histological and biochemical indices in mice. (**A**) Representative hematoxylin and eosin (H&E)-stained tissues from control mice. (**B**,**C**) Representative H&E-stained tissues from PM-D-administered mice, (**B**) local renal tubule atrophy (red arrow), interstitial cell proliferation (green arrow), tubule deposits (blue arrow), and (**C**) tubular epithelial degeneration (red arrow). (**D**) Biochemical indicators of serum renal function were significantly altered after PM-D administration, including creatinine (CREA) and urea. Data are expressed as the mean ± standard deviation (*n* = 6). * *p* < 0.05 compared with the control group.

No mice died after PM-D (2 g/kg) gavage administration for 7 consecutive days. Pathological examinations revealed clear renal cortical and medullary structures and no pathological changes in control mice (Figure 1A). In contrast, renal tubular epithelial cell degeneration, local renal tubular atrophy, and superficial medullary interstitial cell proliferation were observed in experimental mice (Figure 1B,C). Decreased glomerular filtration rates increased CREA and urea levels in these mice (Figure 1D). These observations suggested that PM-D caused renal dysfunction and pathological injury in mice.

### 3.2. Multi-Target Interactions During PM-D Nephrotoxicity

**Figure 2:**
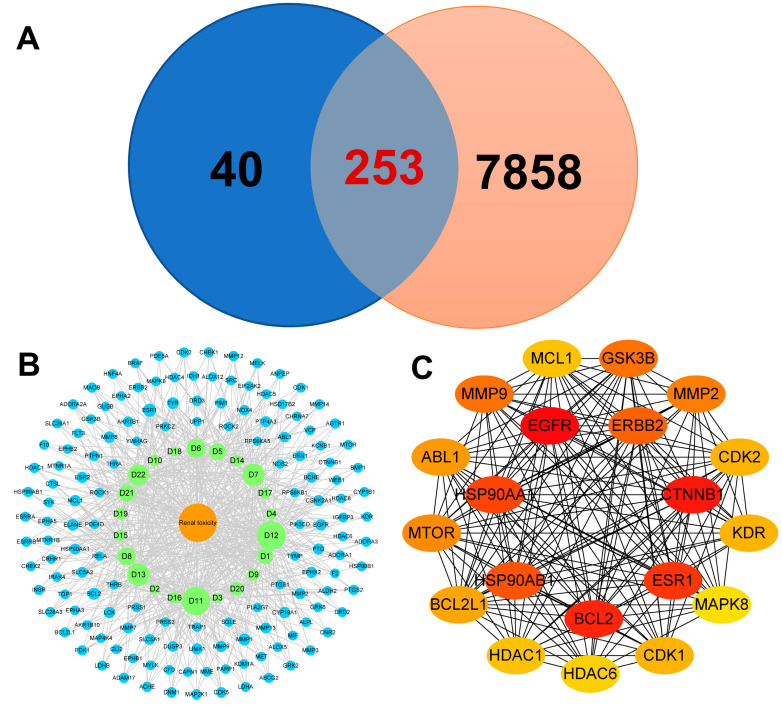
Network pharmacological analysis of PM-D nephrotoxicity. (**A**) A Venn diagram shows predicted PM-D (blue) and nephrotoxic targets (orange), with an intersection representing potential nephrotoxic-associated PM-D targets. (**B**) A “drug component–target–disease” network shows complex multi-target regulation between drug components and disease. Disease is represented by the orange node, drug components by green nodes, and targets by blue nodes. The degree value of a node represents the number of edges that a particular node has with other nodes. Drug component node size is characterized by the degree value; the larger the node, the more critical the drug component in the regulatory network. (**C**) A protein–protein interaction (PPI) network shows interactions between core targets. Proteins are seen as nodes, and interactions between proteins are seen as edges. Node colors reflect the degree value; the darker the color, the higher the degree value, that is, the more critical the protein is in the network.

Network pharmacological analysis was performed based on identified components of PM-D. In our previous study, 27 components were isolated and identified from PM-D, among which anthraquinones and dianthrones were the main toxic compounds that induced acute toxicity in zebrafish embryos [11]. Representative anthraquinone or dianthrone levels in PM-D were previously determined by using HPLC-MS/MS (Table 1) [13]. In the current study, after eliminating components without predicted targets, 293 non-duplicated drug targets were obtained based on 22 components (Table 2) in PM-D. Also, 8111 nephrotoxicity-related disease targets were collected. A Venn diagram showing intersecting targets revealed 253 potential PM-D nephrotoxicity targets, suggesting that most PM-D drug targets were possibly related to nephrotoxicity (Figure 2A). Based on this information, a “drug component–target–disease” network was constructed for interactions with degree values > 2. We observed complex multi-target regulation between drug components and disease, where, for example, N-trans-feruloyl tyramin (D11) and N-trans-feruloyl-3-methyldopamine (D12) had essential roles in nephrotoxicity (Figure 2B). The top 20 targets, ranked by degree value, were then assembled into a PPI network to visualize interactions between targets, from which BCL2, ESR1, CTNNB1, EGFR, and HSP90A proteins were identified as core targets (Figure 2C).

### 3.3. Potential Enrichment Pathways in PM-D Nephrotoxicity

**Figure 3:**
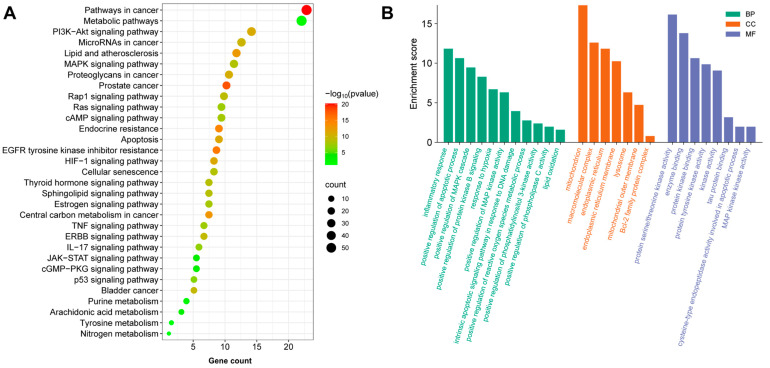
Enrichment analysis of related targets during PM-D nephrotoxicity. (**A**) Kyoto Encyclopedia of Genes and Genomes (KEGG) pathway enrichment analysis is shown as a bubble plot, with bubble size indicating the number of genes in the pathway and colors representing *p* values. (**B**) Gene Ontology (GO) enrichment analysis is shown as a bar diagram including molecular function (MF), cellular component (CC), and biological processes (BP).

Predicted targets were subjected to KEGG enrichment analysis, which showed that PM-D nephrotoxicity mechanisms were mainly related to PI3K-AKT, MAPK, Rap1, Ras, and cAMP signaling pathways, with the PI3K-AKT pathway the most significant (Figure 3A). GO enrichment analysis also showed that biological processes associated with PM-D nephrotoxicity involved inflammatory responses, apoptotic processes, MAPK cascade reactions, and reactive oxygen species (ROS) metabolism, while damaged cellular structures involved mitochondria, lysosomes, and the endoplasmic reticulum (Figure 3B).

### 3.4. Binding Pattern Between Drug Components and Targets

**Figure 4:**
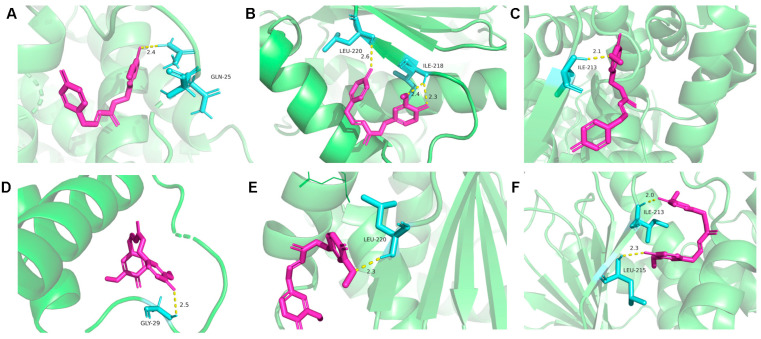
Molecular docking models of D11 and D12 with the key targets BCL2, HSP90A, and HSP90B. (**A**) D11-BCL2. (**B**) D11-HSP90A. (**C**) D11-HSP90B. (**D**) D12-BCL2. (**E**) D12-HSP90A. (**F**) D12-HSP90B. The pink bar structures represent the drug, while the blue structures represent the binding sites of residues in the target proteins. The labels represent the residues and the distance of the interactions between drugs and the target proteins.

Based on the “drug component–target–disease” network, two core components, D11 and D12, were selected for molecular docking validation with key targets BCL2, HSP90A, and HSP90B. The binding activity between docking molecules was estimated by calculating the binding energy (Vina score) using AutoDock1.5.6. When the binding energy is less than −5 kcal/mol, it indicates a good docking state. The findings indicated that two compounds of PM-D had a higher affinity for important targets (binding energies range from −8.54 to −4.76 kcal/mol, Table 3). Among them, the binding of D11 to BCL2 protein showed the best binding activity (−8.54 kcal/mol). The visualization results indicated that the docking point and structure between drug molecule ligands and protein receptors were stable (Figure 4).

### 3.5. Highly Heterogeneous Metabolite Distribution in the Kidney

**Figure 5:**
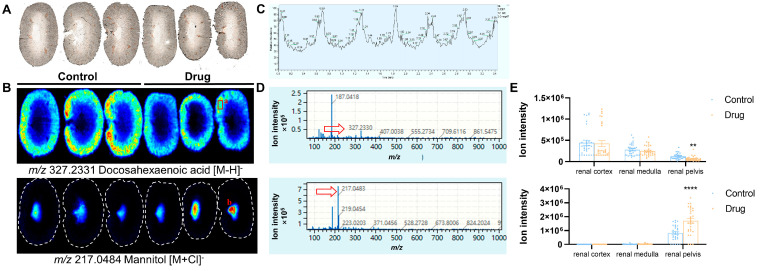
Characterizing heterogeneous metabolite distribution in renal tissue using mass spectrometry imaging. (**A**) An optical scanning image of kidney tissues. (**B**) Representative mass spectrometry images of ion *m/z* 327.2331 and 217.0484. (**C**) Total ion chromatogram of mass spectrometry imaging in negative ion mode. (**D**) Mass spectrum of a single sampling point (a,b in Figure 5B) in the kidney from representative mass spectrometry images. The red arrows indicate the ion peaks of the representative ions *m/z* 327.2331 and 217.0484. (**E**) The average abundance of representative ions *m/z* 327.2331 and 217.0484 in kidney tissue microregions. Data are expressed as the mean ± standard error of the mean. The missing value of the sample is filled with the minimum value within the group. ** *p* < 0.01 and **** *p* < 0.0001 compared with the control group.

The kidney is divided into the renal cortex, renal medulla, and renal pelvis based on structure and function. Optical scanning was performed on the same renal tissue slice before mass spectrometry imaging to visualize morphological characteristics (Figure 5A). Mass spectrometry imaging analysis visualized the spatial structure of renal tissues and retained metabolite in situ information (Figure 5B), with up to tens of thousands of metabolite peaks extracted from a single pixel point and low metabolite abundance still showing clear spatial profiles (Figure 5C,D). Metabolite tissue distribution showed high heterogeneity, e.g., docosahexaenoic acid (*m*/*z* 327.2331, [M − H]^−^) was highly enriched in the renal cortex, while its abundance gradually decreased from this area to the renal pelvis. When compared with control mice, docosahexaenoic acid abundance after PM-D administration decreased, showing a significant difference in the renal medulla (*p* < 0.01). Mannitol (*m*/*z* 217.0484, [M + Cl]^−^) was specifically distributed in the renal medulla, and its abundance significantly increased after PM-D administration (*p* < 0.0001) (Figure 5E).

### 3.6. Metabolic Profile Analyses After PM-D Administration

**Figure 6:**
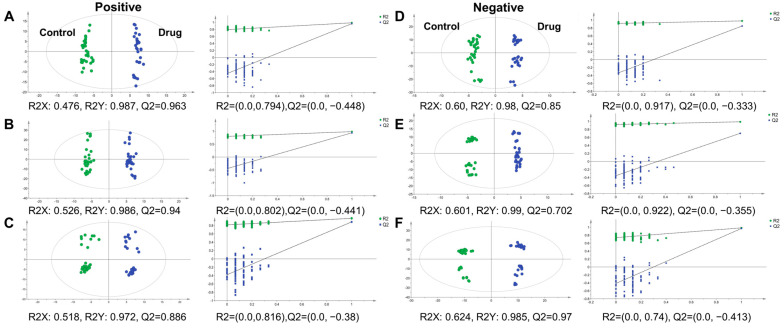
Orthogonal partial least squares discriminant analysis (OPLS-DA) models and permutation tests of mouse kidney tissue microregions. (**A**,**B**) OPLS score plots and permutation test results for the renal pelvis (**A**), renal medulla (**B**), and renal cortex (**C**) in positive ion mode. (**D**–**F**) OPLS score plots and permutation test results for the renal pelvis (**D**), renal medulla (**E**), and renal cortex (**F**) in negative ion mode.

Multivariate statistical analysis was conducted between control and PM-D administration groups to analyze metabolic profile changes. The orthogonal partial least squares discriminant analysis (OPLS-DA) models were constructed by extracting mass spectrometry data from kidney tissue microregions from both groups (Figure 6). According to score plots, metabolites in the renal pelvis, medulla, and cortex showed apparent clustering and grouping trends. To avoid overfitting the OPLS-DA model, permutation tests were used to verify model validity, which showed that all permuted R2 and Q2 values were lower than original values, and the regression line of Q2 points intersected the vertical axis below zero. Differential metabolites were screened according to VIP > 1, *p* < 0.05, and FC > 2 or FC < 0.5 indicators, and imaging results verified. Differential metabolites in positive and negative ion modes were screened and revealed 44 in the renal pelvis, 32 in the renal medulla, and 26 in the renal cortex.

### 3.7. Differential Metabolite Analysis

**Figure 7:**
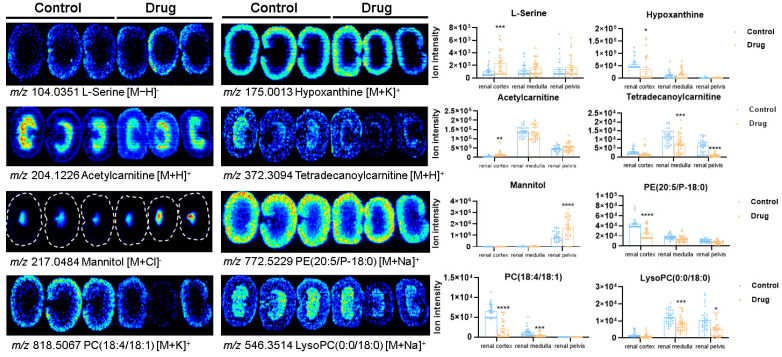
In situ representative differential metabolite distribution and abundance in renal microregions. Data are expressed as the mean ± standard error of the mean. The missing value of the sample is filled with the minimum value within the group. * *p* < 0.05, ** *p* < 0.01, *** *p* < 0.001, and **** *p* < 0.0001 compared with the control group. PC: phosphatidylcholine; PE: phosphatidylethanolamine; LPC: lysophosphatidylcholine.

Identified metabolites included fatty acids, phospholipids, amino acids, organic acids, and nucleosides, with representative biomarkers shown (Table 4). Mass spectrometry imaging revealed spatial heterogeneity in differential metabolite distribution in the kidney, with differential effects on renal microregions after PM-D administration. Amino acids and purine derivatives (e.g., serine and hypoxanthine) were highly distributed in the renal cortex. Also, serine abundance increased, and hypoxanthine abundance decreased in the cortex after PM-D administration. Carnitine metabolites (e.g., acetylcarnitine and tetradecanoylcarnitine) were abundant in the renal medulla. However, tetradecanoylcarnitine was significantly decreased in the renal medulla and renal pelvis, whereas acetylcarnitine was significantly increased in the renal cortex after PM-D administration. The lipid metabolites phosphatidylethanolamine (PE) (20:5/P-18:0) and phosphatidylcholine (PC) (18:4/18:1) were distributed mainly in the renal cortex, whereas lysophosphatidylcholine (LysoPC) (0:0/18:0) was enriched in the renal medulla. Phospholipid metabolite abundance was significantly reduced after PM-D administration (Figure 7).

### 3.8. Differential Metabolic Pathway Analyses

**Figure 8:**
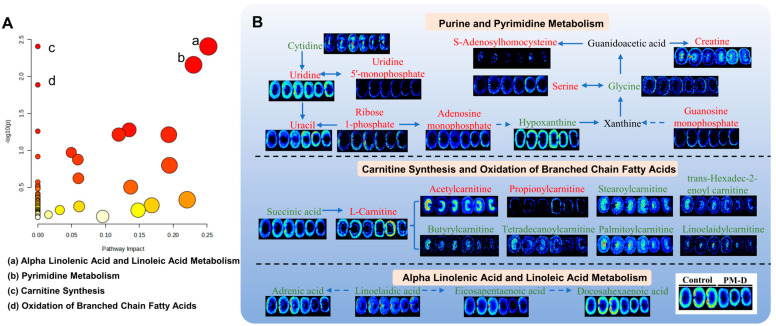
Enrichment analysis of differential metabolic pathways after PM-D-induced nephrotoxicity. (**A**) Enrichment analysis of metabolic pathways related to PM-D-induced nephrotoxicity in The Small Molecule Pathway Database (SMPDB). Metabolic pathways are represented by circles, the darker the color, the smaller the *p* value, and the larger the radius, the larger the enrichment factor. (**B**) Key metabolic pathway changes after PM-D administration by mass spectrometry imaging. Font colors indicate changes in metabolite abundance after PM-D, with red indicating up-regulation and green indicating down-regulation.

Differential metabolites underwent metabolic pathway analysis and showed that after PM-D administration, significantly altered metabolic pathways mainly involved α linolenic and linoleic acid metabolism, pyrimidine metabolism, carnitine synthesis, and oxidation of branched-chain fatty acids (Figure 8A). Abundance changes in key metabolic pathways were further visualized by integrating in situ differential metabolite information (Figure 8B). In pyrimidine metabolism, cytidine, adenosine homocysteine, and creatine were enriched in the renal pelvis, while the remaining metabolites were predominantly distributed in the renal cortex or medulla. Major metabolite abundance increased significantly after PM-D administration. Carnitine was mainly distributed in the renal cortex, whereas acyl-carnitines were predominantly distributed in the renal medulla or renal pelvis. After PM-D administration, carnitine and short-chain acyl-carnitine abundance increased, whereas long-chain acyl-carnitine abundance decreased. Both α-linolenic acid and linoleic acid-related metabolites were primarily distributed in the renal cortex, and their abundance was significantly decreased after PM-D administration.

## 4. Discussion

The kidney is one of the most vulnerable organs to drug damage, and can be damaged during the process of filtration, reabsorption, and excretion of drugs and their metabolites. Improper use of PM can cause not only hepatotoxicity but also some damage to the kidneys. In this study, PM-D was administered to mice at 2 g/kg, equivalent to a human dose of 219.8 mg/kg (13.19 g for 60 kg human), showed potential renal toxicity after 7 days of continuous administration, which potentially degenerated renal tubular epithelial cells and caused local renal tubular atrophy, mild hyperplasia in superficial interstitial cells of the renal medulla, and other pathological changes. Regarding different segments of the renal tubules, the epithelial cells of the proximal tubules, on account of their active reabsorption function and complex cellular structures (such as a large number of microvilli and plasma membrane infoldings), were relatively more vulnerable to damage caused by PM-D and may further undergo tissue atrophy. Meanwhile, the mild hyperplasia in superficial interstitial cells of the renal medulla caused by PM-D mainly affected the thin segment of the renal tubules, altering the osmotic pressure environment around it. In addition, PM-D decreased glomerular filtration rates and damaged renal function, leading to increased serum urea and CREA levels. Such pathological and biochemical index alterations suggested that PM-D-induced nephrotoxicity was not an acute kidney injury but a more insidious disease, causing chronic inflammation and renal insufficiency, and finally chronic kidney disease, which has high incidence rates but a meager informed rate [16]. The Chinese Pharmacopoeia recommends that the clinical dosage of PM is 3–6 g. Traditional toxicity evaluations revealed that PM-induced hepatotoxicity required over 50–70 times the clinical equivalent dose for 4–8 weeks of continuous administration, complicating the toxicity evaluation of herbal medicines [17,18]. The dosage used in this study was higher than that used in the clinic but given that PM accumulation in the kidney may be further elevated with long-term administration or in patients with renal insufficiency, leading to potential toxicity problems. Therefore, nephrotoxic PM-D effects at this dose and related toxicity mechanisms required exploration.

Based on key PM-D components, we predicted drug targets and analyzed nephrotoxicity targets. We observed that *BCL2*, *HSP90A*, *ESR1*, *CTNNB1*, and *EGFR* genes may be potential key PM-D nephrotoxicity targets. Pathway enrichment analysis indicated that PM-D-induced nephrotoxicity may be related to inflammation, apoptosis, and ROS metabolism, with relevant pathways including PI3K-AKT, MAPK, and Rap1 signaling pathways, among which the PI3K-AKT pathway was the most important. When the PI3K-AKT pathway is inhibited, the activity of AKT is reduced, which leads to a concomitant decrease in the activities of the sodium–hydrogen exchanger and the sodium phosphate cotransporter, thereby inducing conspicuous disruptions in the reabsorption function of the proximal tubules [19]. Concurrently, the anti-apoptotic mechanism within the proximal tubule cells is enfeebled. The long-term inhibition of PI3K-AKT signaling pathway may eventually lead to renal tubule atrophy [20], which is consistent with the pathological findings. It was reported that emodin, aloe–emodin, and rhein (PM components) induced apoptosis in renal tubular epithelial cells, suggesting that PM nephrotoxicity was closely related to apoptosis [9]. In our study, molecular docking also demonstrated that the core components, D11 and D12, of PM-D have high affinity for BCL2 and HSP90, which are key proteins in the apoptosis. BCL2 can inhibit caspase activity by inhibiting mitochondrial cytochrome C release or binding to apoptosis-activating factors. The negative regulation of this target induces apoptosis, e.g., p53 forms an inhibitory complex with BCL-2 to induce apoptosis [21,22]. HSP90 can inhibit caspase activation via its interaction with Apaf1 and stabilizes survival signaling proteins, including RIP, Akt, and BCL-2 [23]. PI3K-AKT pathway proteins require HSP90 chaperoning to maintain expression [24]. When HSP90 inhibitors block HSP90/Akt interactions, Akt is dephosphorylated and destabilized, with apoptosis likely increased [25,26]. Estrogen receptor α (ERα) (encoded by *ESR1*) is involved in several physiological processes. When estrogen binds to ERα, it activates PI3K and AKT [27], preventing apoptotic cascades [28]. β-catenin is an adhesion junction protein (encoded by *CTNNB1*) and is a key functional effector downstream of Wnt signaling. This pathway interacts with the PI3K/AKT pathway, e.g., EGF-induced β-catenin depends on AKT activation, and EGFR activation leads to Wnt-free β-catenin activation [29]. Oxidative stress induced by active oxygen metabolism promotes apoptosis, which is implicated in nephrotoxicity [30,31]. For example, aristolochic acid-induced nephrotoxicity promotes apoptosis in renal tubular epithelial cells and is accompanied by increased ROS and oxidative stress responses [32]. The renal toxicity induced by chronic exposure to bisphenol A in mice is also related to oxidative stress and apoptosis [33]. Furthermore, oxidative stress inhibits *BCL2* and *HSP90* expression, which inhibits PI3K/Akt signaling, leading to apoptosis and nephrotoxicity [34].

We also visualized heterogeneous metabolite distribution in the kidney using a spatially resolved metabolomics approach. Enrichment analyses of differential metabolic pathways indicated that nephrotoxicity mechanisms caused by PM-D were closely related to α-oleic acid and linoleic acid metabolism, carnitine biosynthesis, fatty acid oxidation, and pyrimidine metabolism. The kidney is a heterogeneous organ comprising the cortex, medulla, and renal pelvis. All glomeruli are located in the cortex, while renal tubules are distributed in the cortex and medulla. The renal tubule includes the proximal tubule, the thin segment, and the distal tubule. Among them, the proximal tubule is one of the main metabolic centers of the kidney and is mainly located in the renal cortex [35]. As blood flows through the organ, glomeruli filter out macromolecular substances (blood cells, proteins, and fatty acids) while carrying small molecules (metabolic waste and electrolytes) from the blood to the proximal tubule. The proximal tubules have a strong reabsorption capacity and can reabsorb nearly all nutrients (including glucose and amino acids) [36], which is consistent with the fact that the major small molecule metabolites (unsaturated fatty acids, amino acids, and pyrimidine derivatives) characterized by MSI were mainly enriched in the renal cortex. However, mannitol was rarely reabsorbed into the renal tubules after glomerular filtration, so it was specifically enriched in the renal pelvis [37]. After PM-D administration, the abundances of amino acid metabolites in the renal cortex and renal medulla were significantly increased. The incomplete reabsorption of amino acids in the proximal tubule indicated the potential toxic effect of PM-D on the proximal tubule, which might have been closely associated with the interference in the operation of specific amino acid transporters like Slc3a1 and Slc3a2 on the cell membrane [38]. In addition, changes in energy metabolism reflected the damaging effects of PM-D on different tissue structures of the kidney. Glomeruli in the cortex tend to use glucose, for example, renal tubular podocytes, endothelial cells, and mesangial cells, distributed in the glomeruli that mainly rely on glycolysis and use fatty acid oxidation as an alternative energy source when metabolic conditions (e.g., low sugar) change [39,40]. After PM-D administration, up-regulated carnitine and brachylcarnitine levels in the renal cortex may be related to a compensatory increase in fatty acid oxidation due to disordered glycolysis caused by PM-D injury to the glomeruli. The proximal tubules of the renal tubules mainly use fatty acid oxidation to produce ATP, while the thick ascending limbs, and distal convoluted tubules mainly use glucose/pyruvate as energy substrate [41]. After PM-D administration, acylcarnitine derivatives associated with fatty acid oxidation were enriched in the renal medulla and down-regulated, suggesting the mitochondrial damage and the disorder of fatty acid metabolism, further inhibiting the normal operation of ion pumps and transporters dependent on adequate ATP supply, such as sodium and potassium pumps, thus aggravating the dysfunction of renal tubules [42,43]. In serum from patients with chronic kidney disease, saturated and monounsaturated fatty acids were increased, while polyunsaturated fatty acid levels were mostly decreased; however, polyunsaturated fatty acid supplementation inhibited inflammation and oxidative stress and improved renal fibrosis [44,45,46]. This was consistent with our findings showing that PM-D administration down-regulated α-linolenic acid and linoleic acid metabolic pathways. Pyrimidine metabolism disturbances have been widely reported in drug-induced nephrotoxicity, such as those caused by cantharidin and cisplatin, and also triptolide-induced nephrotoxicity [47,48,49]. After PM-D administration, uracil levels and associated intermediates (uridine and uridine 5′-monophosphate) in the renal cortex were significantly up-regulated, and abnormal endogenous purine and pyrimidine metabolism stimulated ROS release to induce oxidative stress.

Using spatially resolved metabolomics, we previously reported that the metabolic mechanisms underpinning hepatotoxicity induced by PM-D were related to linolenic acid, carnitine synthesis, fatty acid oxidation, bile acid synthesis, and purine metabolism processes, and also the tricarboxylic acid cycle, which partially overlapped with the metabolic pathways identified in this study [13]. These results also suggested the interaction of potential hepatorenal toxicity induced by PM-D in vivo, which was related to energy metabolism disorders, lipid metabolism disorders, and nucleotide metabolism imbalance, thereby increasing ROS and exacerbating oxidative stress responses. Network pharmacology further suggested that PM-D nephrotoxicity may have inhibited the PI3K-AKT pathway to induce apoptosis by regulating *BCL2*, *HSP90*, and other targets. These observations will guide further research on PM-D nephrotoxicity mechanisms.

By integrating spatially resolved metabolomics and network pharmacology, the PM-D toxicity study will provide a reference point for hepatorenal toxicity caused by anthraquinone, anthranone, polyphenol compounds, or extracts enriched with these compounds. Our study has some limitations. Firstly, a single dose of PM-D fails to characterize the relationship between drug dose and toxic effects, and the effects of different PM-D doses on nephrotoxicity should be further considered. Secondly, the spatiotemporal metabolic changes caused by PM-D nephrotoxicity in mice over a longer time frame should be considered, which will contribute to a comprehensive understanding of the nephrotoxic effects and potential mechanisms of PM-D. Additionally, PM-D is a traditional Chinese medicine component and has complex targets; thus, its nephrotoxicity mechanisms require further verification in molecular biology studies at the protein and RNA levels. However, our study has highlighted PM-D nephrotoxicity and provides a platform for further PM nephrotoxicity studies.

## 5. Conclusions

PM-D at 2 g/kg for 7 days in mice can induce nephrotoxicity, thus increasing renal biochemical indicators, including urea and CREA, leading to pathological renal changes characterized by local tubular cell epithelium degeneration and tubular atrophy, which can provide a reference for the long-term toxic accumulation of PM. By integrating network pharmacology and spatially resolved metabolomics, potential PM-D nephrotoxic mechanisms were related to oxidative stress caused by metabolic disorders, such as in α-linolenic acid and linoleic acid metabolism, carnitine synthesis, fatty acid oxidation, and pyrimidine metabolism, which potentially caused apoptosis via PI3K-AKT pathway inhibition by regulating BCL2 and HSP90 targets. We provide a preliminary theoretical basis to further examine PM-D nephrotoxicity targets and a platform to study drug-induced renal toxicological mechanisms.

## Figures and Tables

**Table 1:** Four representative toxic components of extract D from *Pleuropterus multiflorus* (PM-D).

Name	Classic	Contents of Analytes (μg/g)
Emodin	Anthraquinones	3989.820
Emodin-8-O-*β*-D-glucopyranoside	Anthraquinones	12,677.423
(*Cis*)-emodin-emodin dianthrones	Dianthrones	1455.940
(*Trans*)-emodin-emodin dianthrones	Dianthrones	1847.708

**Table 2:** Chemical components in PM-D.

No.	Name
D1	(*Cis*)-emodin-emodin dianthrones
D2	(E)-2,3,5,4′-tetrahydroxystilbene-2-O-(2″-O-p-hydroxybenzoyl)-β-D-glucoside
D3	(E)-2,3,5,4′-tetrahydroxystilbene-2-O-*β*-D-(2″-O-monogalloylesters)-
D4	(E)-2,3,5,4′-tetrahydroxystilbene-2-O-*β*-D-(3″-O-monogalloylesters)-
D5	(*Trans*)-emodin-emodin dianthrones
D6	1,3,8-Trihydroxy-6-methyl-10H-anthracen-9-one
D7	Chrysophanol
D8	Emodin
D9	Emodin-6-O-*β*-D-glucopyranoside
D10	Emodin-8-O-*β*-D-glucopyranoside
D11	N-feruloyl tyramin
D12	N-trans-feruloyl-3-methyldopamine
D13	Physcion
D14	Polygonumnolide C1
D15	Polygonumnolide C2
D16	Polygonumnolide C3
D17	Polygonumnolide C4
D18	Polygonumnolide D
D19	Resveratrol-2-O-*β*-D-glucopyranoside
D20	Rhaponticin
D21	Rhein
D22	(*Trans*)-N-caffeoyltyramine

**Table 3:** Target molecule docking results for core components of PM-D.

Core Components	Binding Energy (kcal/mol)		
	BCL2	HSP90A	HSP90B
D11	−8.54	−5.86	−6.15
D12	−8.16	−4.76	−5.75

**Table 4:** Representative endogenous differential metabolites in renal tissue after PM-D administration.

Name	Formula	Ion Adduct	Measured *m/z*	Error × 10^−6^	Fold Change
L-Serine	C_3_H_7_NO_3_	[M − H]^−^	104.0351	−2.11	cortex ↑
Taurine	C_2_H_7_NO_3_S	[M − H]^−^	124.0074	−1.53	pelvis ↑
L-Asparagine	C_4_H_8_N_2_O_3_	[M − H]^−^	131.0462	−2.44	medulla ↑ pelvis ↑
O-Phosphoethanolamine	C_2_H_8_NO_4_P	[M − H]^−^	140.0116	−1.57	cortex ↑ medulla ↑
N-Butyrylglycine	C_6_H_11_NO_3_	[M − H]^−^	144.0666	−1.53	pelvis ↓
L-Valine	C_5_H_11_NO_2_	[M + Cl]^−^	152.0480	−2.50	medulla ↑
Succinic acid	C_4_H_6_O_4_	[M + Cl]^−^	152.9957	−2.03	medulla ↓
L-Phenylalanine	C_9_H_11_NO_2_	[M − H]^−^	164.0715	−1.22	cortex ↑ medulla ↑
N-Acetylleucine	C_8_H_15_NO_3_	[M − H]^−^	172.0976	−1.86	cortex ↓ pelvis ↓
Mannitol	C_6_H_14_O_6_	[M − H]^−^	181.0714	−1.99	medulla ↑ pelvis ↑
Mannitol	C_6_H_14_O_6_	[M + Cl]^−^	217.0484	−1.57	medulla ↑ pelvis ↑
p-Cresol sulfate	C_7_H_8_O_4_S	[M − H]^−^	187.0066	−2.41	medulla ↑ pelvis ↑
D-Mannose	C_6_H_12_O_6_	[M + Cl]^−^	215.0324	−1.81	medulla ↑
Uridine	C_9_H_12_N_2_O_6_	[M + Cl]^−^	279.0389	−0.14	medulla ↓ pelvis ↓
Palmitic acid	C_16_H_32_O_2_	[M + Cl]^−^	291.2096	−0.79	pelvis ↓
Adrenic acid	C_22_H_36_O_2_	[M + Cl]^−^	367.2425	4.28	cortex ↓
Histamine	C_5_H_9_N_3_	[M + H]^+^	112.0865	−3.75	pelvis ↓
Creatine	C_4_H_9_N_3_O_2_	[M + Na]^+^	154.0583	−2.60	cortex ↑
Hypoxanthine	C_5_H_4_N_4_O	[M + K]^+^	175.0013	−2.11	cortex ↓
L-Carnitine	C_7_H_15_NO_3_	[M + K]^+^	200.0679	−2.25	pelvis ↑
Acetylcarnitine	C_9_H_18_NO_4_+	[M + H]^+^	204.1226	−2.11	cortex ↑
Propionylcarnitine	C_10_H_19_NO_4_	[M + H]^+^	218.1380	−3.12	cortex ↑
Butyrylcarnitine	C_11_H_21_NO_4_	[M + Na]^+^	254.1355	−3.07	medulla ↓
Tetradecanoylcarnitine	C_21_H_41_NO_4_	[M + H]^+^	372.3094	−3.84	medulla ↓ pelvis ↓
Palmitoylcarnitine	C_23_H_45_NO_4_	[M + H]^+^	400.3409	−3.07	pelvis ↓
Thiamine	C_12_H_16_N_4_OS	[M + H]^+^	265.1110	−2.87	pelvis ↓
S-Adenosylhomocysteine	C_14_H_20_N_6_O_5_S	[M + H]^+^	385.1305	4.26	pelvis ↑
PC (16:0/16:0)	C_40_H_80_NO_8_P	[M + H]^+^	734.5662	−4.40	pelvis ↓
PC (18:4/18:1)	C_44_H_78_NO_8_P	[M + K]^+^	818.5067	−3.62	cortex ↓ medulla ↓
PE (22:6/P-16:0)	C_43_H_74_NO_7_P	[M + Na]^+^	770.5068	−3.52	cortex ↓
PE (20:5/P-18:0)	C_43_H_76_NO_7_P	[M + Na]^+^	772.5229	−2.93	cortex ↓
PE (16:0/22:6)	C_43_H_74_NO_8_P	[M + K]^+^	802.4762	−2.69	cortex ↓
PE (16:0/20:4)	C_41_H_74_NO_8_P	[M + NH_4_]^+^	757.5520	3.92	medulla ↓
LysoPC (0:0/16:0)	C_24_H_50_NO_7_P	[M + Na]^+^	518.3199	−3.47	pelvis ↓
LysoPC (0:0/18:2)	C_26_H_50_NO_7_P	[M + Na]^+^	542.3203	−2.60	medulla ↓
LysoPC (0:0/18:0)	C_26_H_54_NO_7_P	[M + H]^+^	524.3697	−2.59	cortex ↓ medulla ↓
LysoPC (0:0/18:0)	C_26_H_54_NO_7_P	[M + Na]^+^	546.3514	−2.94	cortex ↓ medulla ↓
LysoPC (0:0/18:0)	C_26_H_54_NO_7_P	[M + K]^+^	562.3255	−2.59	medulla ↓

PC: phosphatidylcholine; PE: phosphatidylethanolamine; LPC: lysophosphatidylcholine; ↑: up-regulation; ↓: down-regulation.

## Data Availability

Dataset is available upon request from the authors.

## References

[1] Xue X., Quan Y., Gong L., Gong X., Li Y. (2020). A review of the processed *Polygonum multiflorum* (Thunb.) for hepatoprotection: Clinical use, pharmacology and toxicology. J. Ethnopharmacol..

[2] Teka T., Wang L., Gao J., Mou J., Pan G., Yu H., Gao X., Han L. (2021). *Polygonum multiflorum*: Recent updates on newly isolated compounds, potential hepatotoxic compounds and their mechanisms. J. Ethnopharmacol..

[3] Rao T., Liu Y.T., Zeng X.C., Li C.P., Ou-Yang D.S. (2021). The hepatotoxicity of *Polygonum multiflorum*: The emerging role of the immune-mediated liver injury. Acta Pharmacol. Sin..

[4] Yu H.S., Wang L.L., He Y., Han L.F., Ding H., Song X.B., Gao X.M., Yun N.R., Li Z. (2020). Advances in the study of the potential hepatotoxic components and mechanism of *Polygonum multiflorum*. Evid. Based Complement. Alternat. Med..

[5] Li H.Z., Duan S.M., Wang Q., Liu Y. (2018). Effects of *Polygonum multiflorum* on kidney injury and renal cell apoptosis in SD rats. Chin. J. Comp. Med..

[6] Huo G.T., Wen H.R., Yang Y.W., Qin C., Wang Q., Mang S.C. (2022). Histopathology of acute kidney injury in SD rats induced by three monomer components of Polygoni Multiflori Radix. Chin. J. Pharmacovigil..

[7] Liu C.Y., Lu L.P., Tian X.Z., Chai Y.H., Xie Y., Zhou W., Niu J.J., Zhang L.Y. (2019). Study on acute hepatorenal toxicity of *Gastrodia gastrodia* powder and freeze-dried powder in mice. Asia-Pac. Tradit. Med..

[8] Wang C., Wu X., Chen M., Duan W., Sun L., Yan M., Zhang L. (2007). Emodin induces apoptosis through caspase 3-dependent pathway in HK-2 cells. Toxicology.

[9] Lan J., Wen H.R., Huang Z.Y., Wang Q., Ma S.C. (2023). Cytotoxicity of HK-2 induced by anthraquinones components of *Polygonum multiflorum*. Chin. J. Pharmacovigil..

[10] Yan Y., Shi N., Han X., Li G., Wen B., Gao J. (2020). UPLC/MS/MS-based metabolomics study of the hepatotoxicity and nephrotoxicity in rats induced by *Polygonum multiflorum* Thunb. ACS Omega.

[11] Yang J.B., Li W.F., Liu Y., Wang Q., Cheng X.L., Wei F., Wang A.G., Jin H.T., Ma S.C. (2018). Acute toxicity screening of different extractions, components and constituents of *Polygonum multiflorum* Thunb. on zebrafish (*Danio rerio*) embryos in vivo. BioMed Pharmacother..

[12] Li H.Y., Yang J.B., Li W.F., Qiu C.X., Hu G., Wang S.T., Song Y.F., Gao H.Y., Liu Y., Wang Q. (2020). In vivo hepatotoxicity screening of different extracts, components, and constituents of *Polygoni multiflori* Thunb. in zebrafish (*Danio rerio*) larvae. Biomed. Pharmacother..

[13] Jiang H.Y., Gao H.Y., Li J., Zhou T.Y., Wang S.T., Yang J.B., Hao R.R., Pang F., Wei F., Liu Z.G. (2022). Integrated spatially resolved metabolomics and network toxicology to investigate the hepatotoxicity mechanisms of component D of *Polygonum multiflorum* Thunb. J. Ethnopharmacol..

[14] Li X., Liu Z., Liao J., Chen Q., Lu X., Fan X. (2023). Network pharmacology approaches for research of traditional Chinese medicines. Chin. J. Nat. Med..

[15] Zhao L., Zhang H., Li N., Chen J., Xu H., Wang Y., Liang Q. (2023). Network pharmacology, a promising approach to reveal the pharmacology mechanism of Chinese medicine formula. J. Ethnopharmacol..

[16] Shah S.V., Baliga R., Rajapurkar M., Fonseca V.A. (2007). Oxidants in chronic kidney disease. J. Am. Soc. Nephrol..

[17] Li C.Y., Tu C., Gao D., Wang R.L., Zhang H.Z., Niu M., Li R.Y., Zhang C.E., Li R.S., Xiao X.H. (2016). Metabolomic study on idiosyncratic liver injury induced by different extracts of *Polygonum multiflorum* in rats integrated with pattern recognition and enriched pathways analysis. Front Pharmacol..

[18] Wang T., Wang J.Y., Jiang Z.Z., Zhou Z.X., Li Y.Y., Zhang L., Zhang L.Y. (2012). Study on hepatotoxicity of aqueous extracts of *Polygonum multiforum* in rats after 28-day oral administration-analysis on correlation of cholestasis. Zhongguo Zhong Yao Za Zhi.

[19] Satoh N., Nakamura M., Suzuki M., Suzuki A., Seki G., Horita S. (2015). Roles of Akt and SGK1 in the regulation of renal tubular transport. BioMed Res. Int..

[20] Wang H.S., Gao L.J., Zhao C.C., Fang F., Liu J.Z., Wang Z., Zhong Y., Wang X.T. (2024). The role of PI3K/Akt signaling pathway in chronic kidney disease. Int. Urol. Nephrol..

[21] Wei H., Wang H., Wang G., Qu L., Jiang L., Dai S., Chen X., Zhang Y., Chen Z., Li Y. (2023). Structures of p53/BCL-2 complex suggest a mechanism for p53 to antagonize BCL-2 activity. Nat. Commun..

[22] Hemann M.T., Lowe S.W. (2006). The p53-Bcl-2 connection. Cell Death Differ..

[23] Hoter A., El-Sabban M.E., Naim H.Y. (2018). The HSP90 family: Structure, regulation, function, and implications in health and disease. Int. J. Mol. Sci..

[24] Giulino-Roth L., van Besien H.J., Dalton T., Totonchy J.E., Rodina A., Taldone T., Bolaender A., Erdjument-Bromage H., Sadek J., Chadburn A. (2017). Inhibition of Hsp90 suppresses PI3K/AKT/mTOR signaling and has antitumor activity in Burkitt Lymphoma. Mol. Cancer Ther..

[25] Lanneau D., de Thonel A., Maurel S., Didelot C., Garrido C. (2007). Apoptosis versus cell differentiation: Role of heat shock proteins HSP90, HSP70 and HSP27. Prion.

[26] Wu J., Li L., Wang Y., Ren X., Lin K., He Y. (2019). The HSP90/Akt pathway may mediate artemether-induced apoptosis of Cal27 cells. FEBS Open Bio..

[27] Guo Y., Wu Y., Huang T., Huang D., Zeng Q., Wang Z., Hu Y., Liang P., Chen H., Zheng Z. (2024). Licorice flavonoid ameliorates ethanol-induced gastric ulcer in rats by suppressing apoptosis via PI3K/AKT signaling pathway. J. Ethnopharmacol..

[28] Straub R.H. (2007). The complex role of estrogens in inflammation. Endocr. Rev..

[29] Jere S.W., Abrahamse H., Houreld N.N. (2023). Interaction of the AKT and β-catenin signalling pathways and the influence of photobiomodulation on cellular signalling proteins in diabetic wound healing. J. Biomed. Sci..

[30] Liu X.Y., Zhang F.R., Shang J.Y., Liu Y.Y., Lv X.F., Yuan J.N., Zhang T.T., Li K., Lin X.C., Liu X. (2018). Renal inhibition of miR-181a ameliorates 5-fluorouracil-induced mesangial cell apoptosis and nephrotoxicity. Cell Death Dis..

[31] Ho H.J., Shirakawa H. (2022). Oxidative stress and mitochondrial dysfunction in chronic kidney disease. Cells.

[32] Liu M.C., Maruyama S., Mizuno M., Morita Y., Hanaki S., Yuzawa Y., Matsuo S. (2003). The nephrotoxicity of Aristolochia manshuriensis in rats is attributable to its aristolochic acids. Clin. Exp. Nephrol..

[33] Tang Z., Wang H., Zhang Z., Kong Y., Lei X., Yuan J. (2023). Mechanism of nephrotoxicity induced by chronic exposure of bisphenol A in mice based on oxidative stress and cell apoptosis. Chin. J. Biotechnol..

[34] Wang C., Feng L., Ma L., Chen H., Tan X., Hou X., Song J., Cui L., Liu D., Chen J. (2017). Alisol A 24-acetate and alisol B 23-acetate induced autophagy mediates apoptosis and nephrotoxicity in human renal proximal tubular cells. Front. Pharmacol..

[35] Gantsova E., Serova O., Vishnyakova P., Deyev I., Elchaninov A., Fatkhudinov T. (2024). Mechanisms and physiological relevance of acid-base exchange in functional units of the kidney. PeerJ..

[36] Ho K.M., Morgan D.J.R. (2022). The proximal tubule as the pathogenic and therapeutic target in acute kidney injury. Nephron.

[37] Nissenson A.R., Weston R.E., Kleeman C.R. (1979). Mannitol. West. J. Med..

[38] Chrysopoulou M., Rinschen M.M. (2024). Metabolic rewiring and communication: An integrative view of kidney proximal tubule function. Annu. Rev. Physiol..

[39] Wen L., Li Y., Li S., Hu X., Wei Q., Dong Z. (2021). Glucose metabolism in acute kidney injury and kidney repair. Front. Med..

[40] Singh P. (2023). Reprogramming of energy metabolism in kidney disease. Nephron.

[41] Clark A.J., Parikh S.M. (2020). Mitochondrial Metabolism in Acute Kidney Injury. Semin. Nephrol..

[42] Li Y., Gu W., Hepokoski M., Pham H., Tham R., Kim Y.C., Simonson T.S., Singh P. (2023). Energy metabolism dysregulation in chronic kidney disease. Kidney360.

[43] Cheng Y.W., Cheng C.J. (2024). Mitochondrial bioenergetics: Coupling of transport to tubular mitochondrial metabolism. Curr. Opin. Nephrol. Hypertens..

[44] Han S., Choi H., Park H., Kim J.J., Lee E.J., Ham Y.R., Na K.R., Lee K.W., Chang Y.K., Choi D.E. (2023). Omega-3 fatty acids attenuate renal fibrosis via AMPK-mediated autophagy flux activation. Biomedicines.

[45] An W.S., Kim H.J., Cho K.H., Vaziri N.D. (2009). Omega-3 fatty acid supplementation attenuates oxidative stress, inflammation, and tubulointerstitial fibrosis in the remnant kidney. Am. J. Physiol. Ren. Physiol..

[46] Gai Z., Wang T., Visentin M., Kullak-Ublick G.A., Fu X., Wang Z. (2019). Lipid accumulation and chronic kidney disease. Nutrients.

[47] He T., Xiong L., Zhang Y., Yan R., Yu M., Liu M., Liu L., Duan C., Li X., Zhang J. (2023). Mice kidney biometabolic process analysis after cantharidin exposure using widely-targeted metabolomics combined with network pharmacology. Food Chem. Toxicol..

[48] Xie L., Zhao Y., Duan J., Fan S., Shu L., Liu H., Wang Y., Xu Y., Li Y. (2020). Integrated proteomics and metabolomics reveal the mechanism of nephrotoxicity induced by triptolide. Chem. Res. Toxicol..

[49] Xu L., Zhang Y., Zhang P., Dai X., Gao Y., Lv Y., Qin S., Xu F. (2019). Integrated metabolomics and network pharmacology strategy-driven active traditional Chinese medicine ingredients discovery for the alleviation of cisplatin nephrotoxicity. Chem. Res. Toxicol..

